# Evaluating sentiment analysis models: A comparative analysis of vaccination tweets during the COVID-19 phase leveraging DistilBERT for enhanced insights

**DOI:** 10.1016/j.mex.2025.103407

**Published:** 2025-05-30

**Authors:** Renuka Agrawal, Mehuli Majumder, Ishita Yadav, Nandini Taneja, Safa Hamdare, Preeti Hemnani

**Affiliations:** a Symbiosis Institute of Technology – Pune Campus, Symbiosis International (Deemed University), Pune, India; b Nottingham Trent University-Cliffton Campus, Nottingham, UK; c Department of Electronics and Telecommunication Engineering, SIES Graduate School of Technology, Mumbai, India

**Keywords:** Machine learning, Sentiment analysis, COVID-19, Vaccination, Natural Language processing, *DistilBERT Sentiment Analysis*

## Abstract

This study investigates public sentiment toward COVID-19 vaccinations by analyzing Twitter data using advanced machine learning (ML) and natural language processing (NLP) techniques. Recognizing social media as a valuable source for gauging public opinion during health crises, the research aims to inform policies on content moderation and misinformation control.

• Comparative Analysis of Embedding Techniques and ML Models: The study evaluates two embedding techniques—TF-IDF and Word2Vec—across five ML models: LinearSVC, Random Forest, Gradient Boosting Machine (GBM), XGBoost, and AdaBoost.
• The models were tested using two training-testing splits (70–30 and 80–20) to assess their performance on noisy, unlabeled, and imbalanced sentiment data.
• Utilization of DistilBERT for Pseudo-Labeling: To enhance labeling accuracy, DistilBERT was employed for pseudo-labeling, capturing semantic nuances often missed by traditional ML techniques. This approach enabled more effective sentiment classification of tweets.

The findings underscore the effectiveness of automated annotation, hybrid modeling, and embedding strategies in analyzing unstructured social media data. Such approaches provide valuable insights for public health applications, particularly in understanding vaccine hesitancy and shaping communication strategies. The study highlights the potential of integrating advanced NLP techniques to better comprehend and respond to public sentiments during pandemics or similar emergencies.

## Graphical abstract

**Figure:**
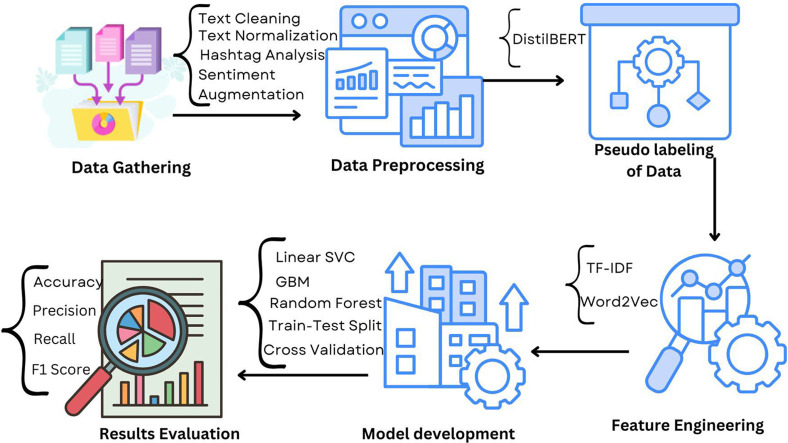

Specifications table

**Table:** 

Subject area:	Computer Science
More specific subject area:	*Machine Learning*
Name of the reviewed methodology:	*DistilBERT Sentiment Analysis*
Keywords:	*Machine Learning, Sentiment Analysis, DistilBERT, COVID-19, Vaccination*
Resource availability:	*Vaccination tweets from Kaggle*
Review question:	• *What role does DistilBERT play in pseudo-labeling, and how does it enhance the analysis of noisy, unlabeled data?* • *How do TF-IDF and Word2Vec differ in analyzing social media sentiment data?* • *How can insights from this study inform healthcare policy and communication strategies, particularly concerning vaccination sentiment?*

## Background

The COVID-19 pandemic led to a surge in social media activity, with platforms like Twitter becoming pivotal in shaping public discourse around vaccination. This increase in digital conversations underscored the importance of understanding public sentiment, as these discussions significantly influenced health communication strategies, public behaviors, and policymaking [1]. Existing research in this domain highlights the relevance of sentiment analysis using various computational techniques. Studies like those by Rustam et al. and Qorib et al. have employed traditional machine learning models, such as Random Forest and Support Vector Machines (SVM), with feature extraction methods like Bag-of-Words (BoW) and TF-IDF to classify sentiments in COVID-19-related tweets [2,3]. However, these conventional approaches often fail to capture the semantic nuances present in textual data, especially in noisy, informal social media environments [4].

To address these limitations, this research integrates state-of-the-art techniques with traditional machine learning models, offering a robust framework for sentiment analysis. Transformer-based models like DistilBERT are employed for pseudo-labeling, enabling the extraction of contextual embeddings that capture subtle semantic relationships in tweets [5,6]. These embeddings are combined with traditional features such as TF-IDF or Word2Vec, creating a hybrid approach that balances modern and classical methodologies. Unlike earlier studies that relied on static labeled datasets [7,8], this study incorporates advanced preprocessing techniques like tokenization, lemmatization, and domain-specific feature engineering. These steps enrich the dataset by adding contextual information, such as hashtag analysis and VADER sentiment scores, ensuring a comprehensive representation of social media sentiment [9].

A unique aspect of this study is its focus on neutral sentiment classification, a challenge often overlooked in sentiment analysis research. Neutrality is inherently difficult to identify due to its subtle and context-dependent nature. This research introduces a calibrated threshold for neutrality, improving the accuracy of distinguishing mixed or ambiguous sentiments. This innovative approach addresses gaps in prior studies, such as those by Kiritchenko et al. and Socher et al., which primarily focused on binary or ternary sentiment classifications [10,11].

The integration of diverse tools and methodologies further distinguishes this work. DistilBERT provides a foundation for capturing deep semantic meaning, while tools like TF-IDF emphasize term-specific features, and Word2Vec identifies semantic relationships between words [12,13]. VADER complements these techniques by adding a rule-based layer for detecting straightforward emotional cues, ensuring a comprehensive analysis. This cohesive pipeline outperforms traditional sentiment analysis methods, as demonstrated in earlier studies on social media sentiment during health crises, such as those by Medhat et al. and Zhang et al. [14,15].

Despite the widespread use of machine learning techniques for sentiment analysis, challenges remain in handling noisy, unlabeled social media data and effectively classifying neutral sentiments. Existing studies often overlook the benefits of hybrid approaches combining transformer models and traditional feature extraction methods. By combining advanced natural language processing techniques with traditional machine learning tools, this research offers a significant advancement in sentiment analysis. It demonstrates how such hybrid approaches can better analyze large-scale, noisy datasets like those from Twitter, providing actionable insights for public health communication during crises like the COVID-19 pandemic [16,17].

## Methodology

**Fig. 1:**
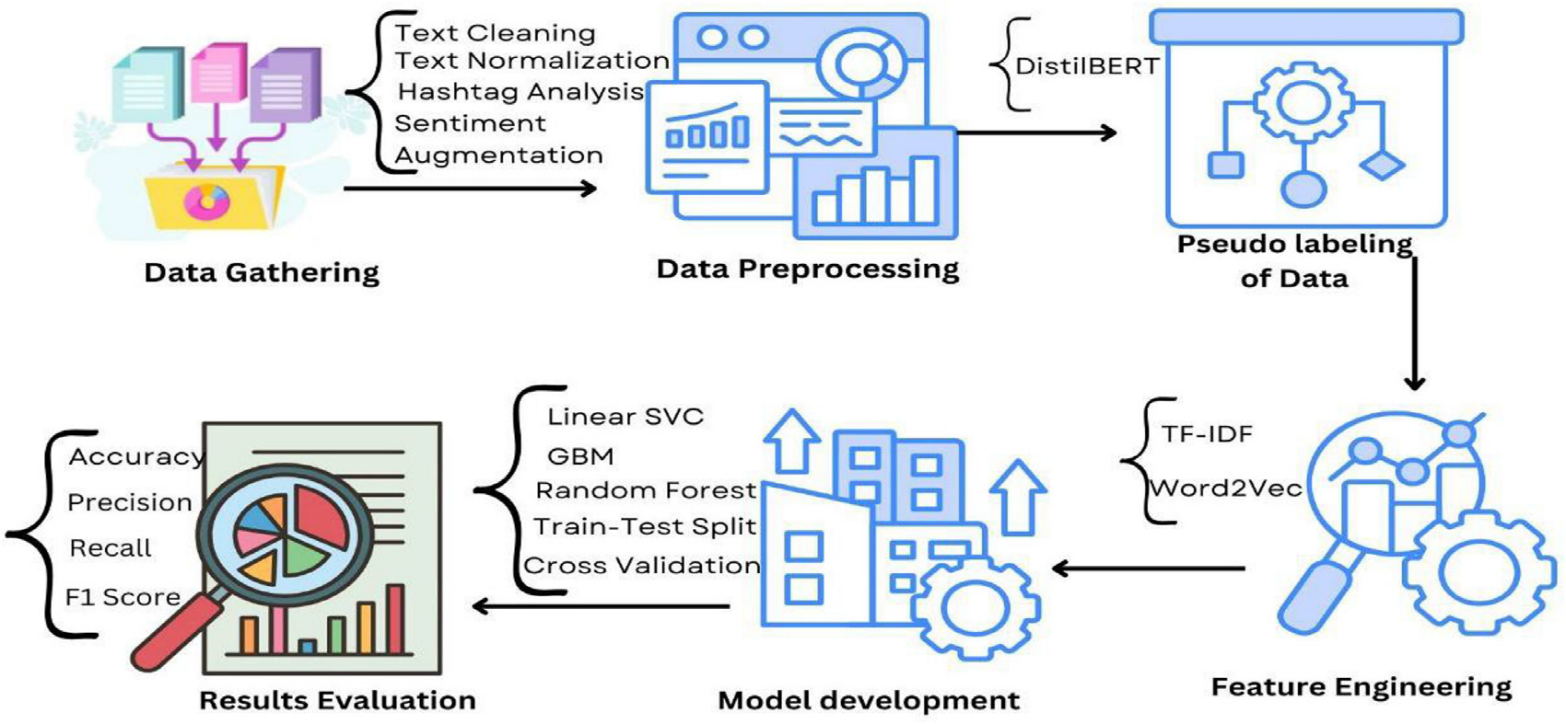
Graphical abstract of proposed methodology.

The dataset used in this study, vaccination_tweets.csv, was sourced from Kaggle and comprises 10,933 tweets with 19 columns for each tweet. Fig. 1 shows Graphical Abstract for Proposed Methodology.

**Fig. 2:**
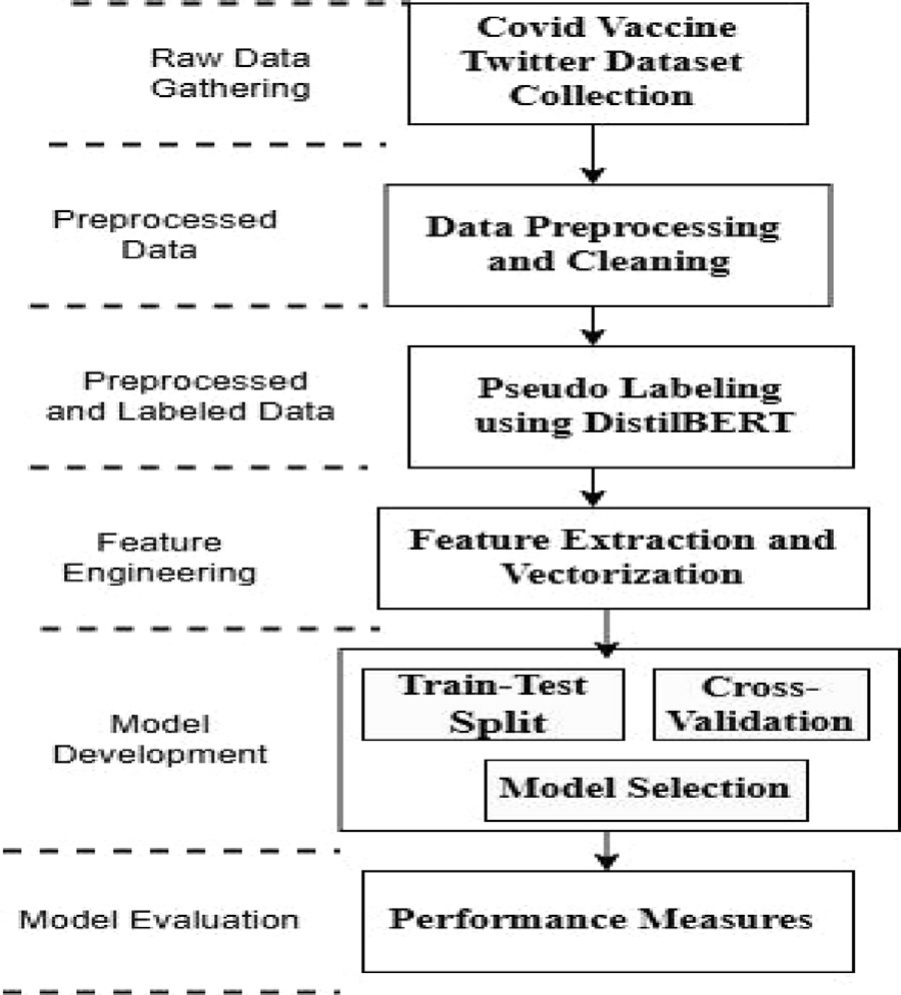
Logical flow of methodology.

Fig. 2 enlists the logical flow of methodology adopted for sentiment classification. The steps involved in sentiment analysis are covered in detail in subsequent sections.

### Dataset preprocessing

**Fig. 3:**
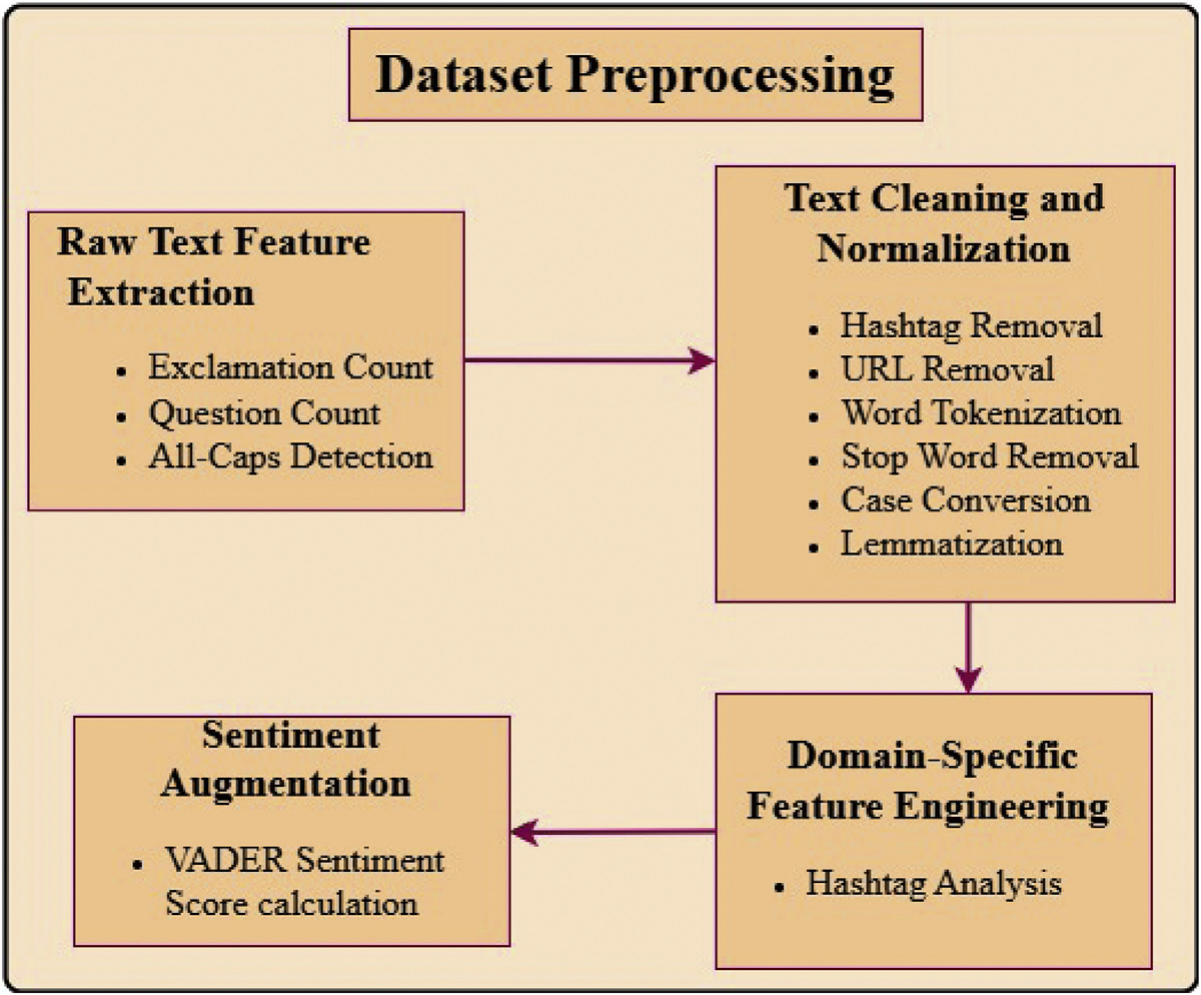
Steps involved in dataset preprocessing.

The dataset used in this study, vaccination_tweets.csv, was sourced from Kaggle and comprises 10,933 tweets samples with each sample having 23 attributes. Relevant attributes were chosen and other such as User_ID, User_Name, User_description and others nonrelevant features were trimmed. However, features of raw text such as exclamation _count were retained. The dataset underwent extensive preprocessing and data cleaning to enhance its quality and reliability. In the context of vaccination sentiment analysis research, effective text preprocessing was critical for ensuring the quality and relevance of the data used in model training and evaluation. The steps involved for data preprocessing are included in Fig. 3.

The preprocessing steps undertaken are detailed below:

• **Raw Text Feature Extraction:** To capture various tonal elements in tweets, several features were analyzed. The number of exclamation marks (!) was counted to identify expressions of excitement or emphasis, while the number of question marks (?) was recorded to detect inquisitive or uncertain tones. Additionally, tweets containing fully capitalized words were flagged, as these often signify strong emotions or emphasis.
• **Text Cleaning and Normalization:** Several preprocessing steps were applied to normalize and clean the textual data.
  √ First, all text was converted to lowercase to eliminate case sensitivity.
  √ User mentions (e.g., @username) and hashtags (e.g., #keyword) were removed, with hashtags analyzed separately to avoid redundancy.
  √ Hyperlinks were stripped to focus on textual content,
  √ non-alphabetic characters were excluded during word tokenization to retain only meaningful linguistic elements.
  √ Common stop words, such as "and" and "the," were removed to enhance the focus on significant words.
  √ Finally, lemmatization was performed using part-of-speech tagging to reduce words to their base forms, ensuring semantic consistency and uniformity [18].
• **Domain-Specific Feature Engineering:** Hashtag analysis involves storing hashtags in a separate column and identifying domain-specific keywords related to vaccination, such as "VaccinesWork" and "CovidVaccine." Tweets containing these keywords were flagged as having vaccination-related hashtags to facilitate focused analysis.

**Fig. 4:**
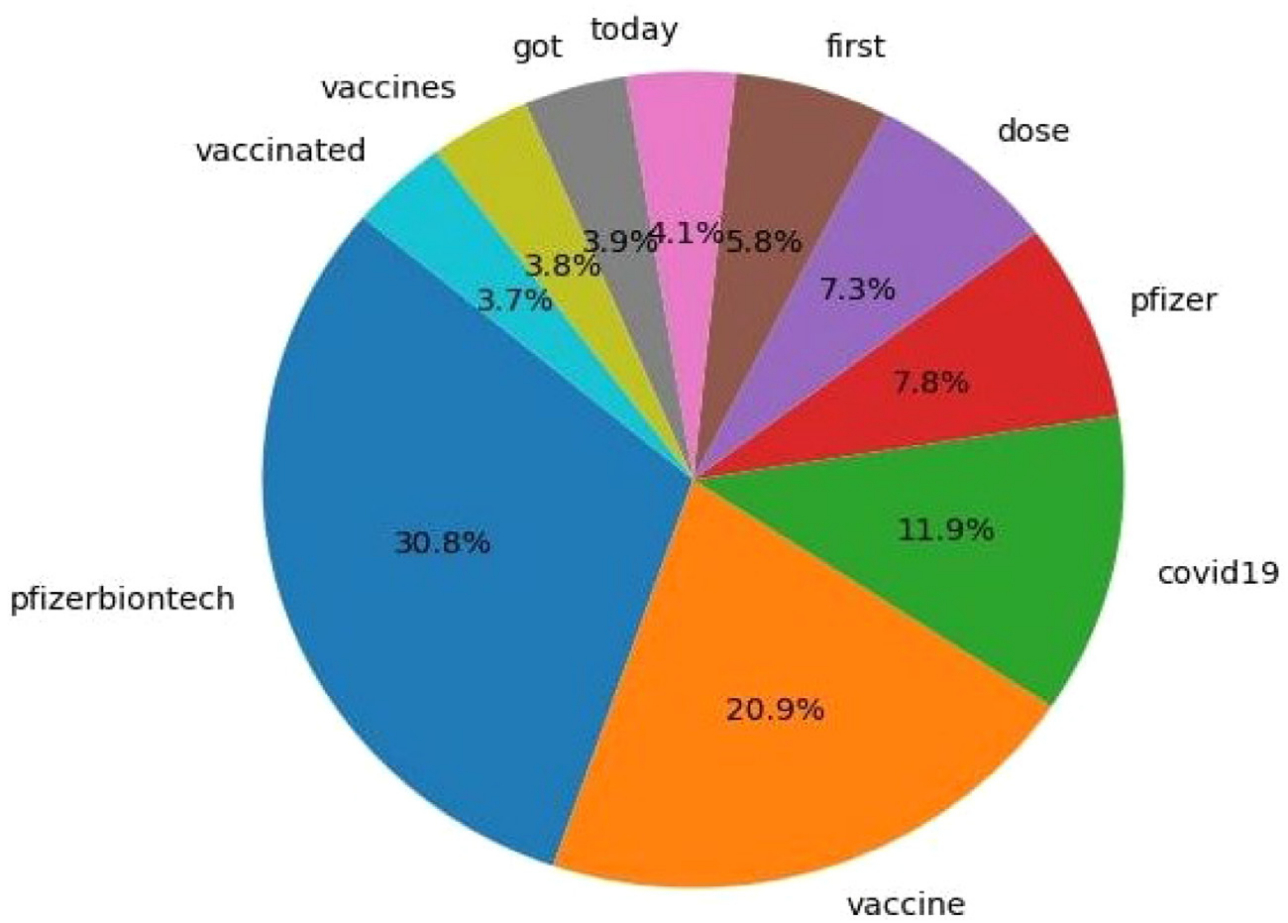
Top 10 most frequent words in tweets.

Fig. 4 presents the Top 10 Most Frequent Words in the preprocessed vaccination tweets dataset, reflecting key themes in public discussions surrounding COVID-19 vaccination. The word "pfizerbiontech" (30.8 %) appears most frequently, highlighting significant attention toward the Pfizer-BioNTech vaccine. Similarly, "vaccine" (20.9 %) and "covid19″ (11.9 %) emphasize the central focus on vaccines and the pandemic as the driving factor behind these conversations. Words such as "pfizer" (7.8 %), "dose" (7.3 %), and "first" (5.8 %) further underscore discussions about specific vaccines and vaccination schedules, particularly the administration of doses. Additionally, terms like "today" (4.1 %), "got" (3.9 %), and "vaccinated" (3.7 %) reflect personal narratives and real-time updates on vaccination experiences. The frequent occurrence of these words results from effective text preprocessing that includes text cleaning and normalization, which retained significant words, and domain-specific feature engineering, which preserved keywords related to vaccination. The insights gained from this analysis, combined with sentiment models like VADER, help capture public perceptions and emotional tones, contributing to a deeper understanding of vaccination-related discourse on social media.

• **Sentiment Augmentation Using VADER:** The VADER sentiment analysis tool was utilized to calculate the compound sentiment score for each tweet, offering a quick estimate of overall sentiment polarity—positive, negative, or neutral. This score was added as a distinct feature in the dataset. VADER was specifically chosen for its ability to handle informal text elements such as emoticons, slang, and acronyms, which are common in social media. Its extensive lexicon of over 7500 words is annotated with sentiment intensities that reflect online usage, assigning scores to words like “awesome” (positive), “terrible” (negative), and “okay” (neutral).

VADER employs a combination of lexical dictionaries and contextual rules to determine sentiment intensity [19]. These rules account for modifiers (e.g., “very” or “extremely”) that amplify or dampen sentiment, punctuation (e.g., exclamation marks) that enhance sentiment strength, capitalization for emphasis, and negation (e.g., “not” or “never”) that reverses sentiment. This lightweight and context-aware approach makes VADER particularly suited for analyzing brief, informal social media text.

In this study, VADER sentiment scores were incorporated as an additional feature, complementing other models like DistilBERT, LinearSVC, and LSTM. While these data-driven models excel at identifying complex patterns, they may overlook straightforward sentiment cues that VADER captures effectively. This integration of VADER scores enhances model robustness by introducing a rule-based perspective to sentiment analysis, improving the ability to detect emotional tones and subtleties in social media content.

Additionally, TextBlob was also tested for calculating polarity scores. However, its rule-based nature proved less effective as it struggled to adapt to new data, frequently returning a neutral score of zero for unfamiliar inputs. Overall, these preprocessing steps, including VADER sentiment analysis, ensured the dataset was cleaned, normalized, and enriched with linguistic and domain-specific features, thus enhancing the robustness and accuracy of subsequent sentiment analysis tasks [20,21].

### Labelling preprocessed data

Sentiment classification was conducted by processing each tweet through a pipeline that returned a sentiment label, such as "POSITIVE," "NEGATIVE," or "NEUTRAL." The core of this sentiment analysis process was the DistilBERT base uncased model, fine-tuned on the Stanford Sentiment Treebank (SST-2) dataset. DistilBERT, a distilled version of the BERT transformer model, was selected for its ability to retain much of BERT's accuracy while being significantly faster and more lightweight [22]. Known for its effectiveness in sentiment classification tasks, DistilBERT can identify nuanced patterns and capturing subtle emotional cues in text [23]. The sentiment analysis pipeline was implemented in Python, utilizing Hugging Face's pipeline API to ensure seamless integration and streamlined workflow. The key steps included:

○ **Loading the Dataset:** The preprocessed dataset of vaccination tweets was loaded using pandas, preparing it for analysis. Each tweet's text served as input to the sentiment classification pipeline.
○ **Sentiment Classification:** The Hugging Face sentiment-analysis pipeline was initialized with the DistilBERT model, which predicted sentiment labels (e.g., "POSITIVE", "NEGATIVE") and provided confidence scores. To address ambiguous or mixed sentiments, a neutral threshold was applied. If the model’s confidence score for a prediction was below 0.6, the tweet was labeled as "NEUTRAL." This threshold was calibrated to capture subtle or uncertain sentiments effectively, ensuring a balanced sentiment distribution.
○ **Handling Missing Data:** Tweets with missing or invalid text were directly labeled as "NEUTRAL" to maintain consistency in the dataset. Any remaining rows with missing sentiment labels were removed during the final data cleaning process.
○ **Saving the Labeled Dataset:** The classified dataset, now enriched with sentiment labels, was saved as vaccination_tweets_labeled.csv. This labeled dataset was critical for the subsequent stages of the project.

After labeling the tweets, the labeled dataset was saved for use in training various machine learning algorithms, including LinearSVC, Random Forest, and GBM. This dataset served as the foundation for evaluating and comparing the performance of these models, ensuring consistency and reliability throughout the project. By integrating the powerful DistilBERT model, an effectively calibrated neutral threshold, and meticulous data handling, the sentiment analysis pipeline achieved high accuracy and robustness [24]. This approach not only ensured precise sentiment classification but also facilitated the creation of a high-quality labeled dataset crucial for subsequent machine learning experimentation.

### Model development/designing training

To evaluate the performance of various models in our vaccination sentiment analysis study, two feature extraction techniques—TF-IDF and Word2Vec embeddings—were implemented and compared across five machine learning models: LinearSVC, Random Forest, GBM, XGBoost and AdaBoost. Each model was trained and validated using these features, allowing for a comprehensive assessment of their predictive capabilities.

a) **Dataset Preparation:** The dataset consisted of labeled tweets, with a 'text' column containing tweet content and a 'sentiment' column representing sentiment labels. Rows with missing text data were removed to maintain the integrity of the analysis.
b) **Feature Extraction:**
  • **TF-IDF:** A TF-IDF vectorizer was used to convert tweet text into numerical feature vectors, with the maximum number of features capped at 5000 to balance computational efficiency and feature richness.
  • **Word2Vec:** A pre-trained Word2Vec model generated word embeddings for each tweet. The average of the Word2Vec embeddings of the constituent words was computed for each tweet. Words not found in the model's vocabulary were replaced with zero vectors.
c) **Cross-Validation:** A 5-fold cross-validation strategy was employed, where the dataset was split into five subsets. In each iteration, four subsets were used for training, and the fifth subset was used for validation. This process ensured a fair comparison between the TF-IDF and Word2Vec feature extraction methods across different models, providing a robust evaluation of their effectiveness in sentiment classification [25]. The StratifiedKFold method preserved the class distribution in each fold, reducing the risk of overfitting and ensuring reliable evaluation. Cross-validation offers several advantages:
  • It provides a more robust estimate of model performance by averaging results across diverse subsets of data.
  • It reduces the risk of overfitting by evaluating models on different parts of the dataset.
d) **Train-Test Splits:** After cross-validation, the dataset was split into training and testing subsets using 70–30 and 80–20 splits. These splits offered additional evaluation metrics on unseen data, further ensuring the consistency of model performance. Models were trained on the training subset and evaluated on the test subset to compute accuracy.
e) **Model Training and Pipeline Setup:** The models for each feature extraction technique were trained using pipelines. For LinearSVC, the pipeline included the feature extraction step (either TF-IDF or Word2Vec) followed by the LinearSVC classifier with balanced class weights to handle potential class imbalances. A fixed random state of 42 was used for reproducibility. Similar pipelines were created for Random Forest and GBM models, with hyperparameters fine-tuned for optimal performance.
f) **Model Saving:** The trained models, after cross-validation and the specific train-test splits, were saved as pickle files for reproducibility and future analysis.

### Deployment

An application was developed using Flask to provide a web interface for sentiment analysis. The application allows users to input tweet text and receive predictions on whether the sentiment is positive or negative, making it a practical tool for real-time sentiment evaluation. After evaluating various configurations and data splits, the Linear Support Vector Classifier (SVC) combined with TF-IDF embeddings was chosen for final deployment, as the 80–20 split model achieved the highest performance accuracy, making it the optimal choice for deployment.

## Model evaluation

**Fig. 5:**
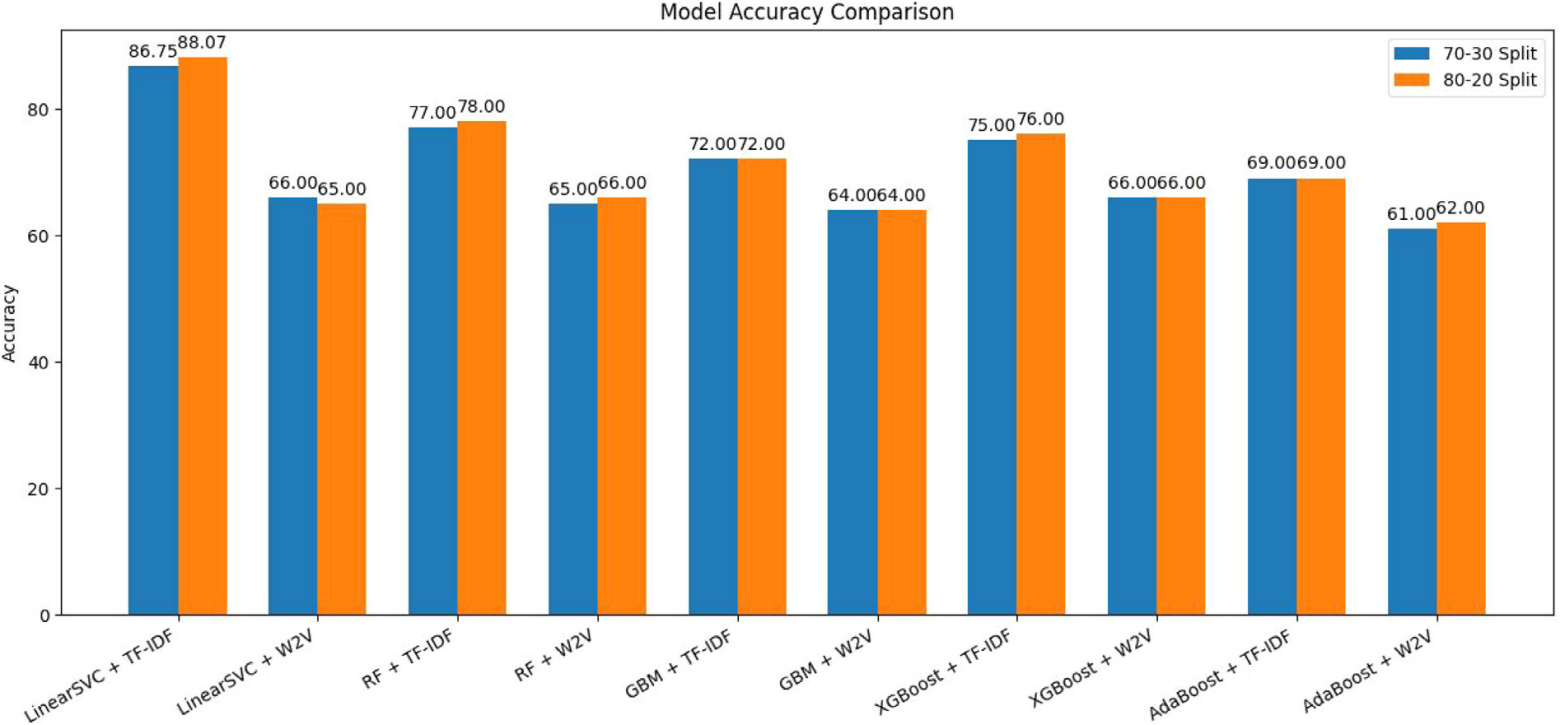
Comparative analysis of model accuracy for sentiment classification.

The sentiment analysis models were successfully trained and deployed, enabling users to input tweet text and receive real-time sentiment classifications as "POSITIVE," "NEGATIVE," or "NEUTRAL." This functionality offers valuable insights into public sentiment toward vaccinations, which can help inform public health communication strategies and enhance understanding of community concerns regarding vaccines [25]. To evaluate the performance of the models, key metrics such as accuracy, precision, recall, and F1-score were calculated using a confusion matrix. The confusion matrix provides a visual representation of the models' performance across different sentiment classes, allowing for a comprehensive analysis. By examining these metrics alongside the confusion matrix, one can gain a deeper understanding of the strengths and weaknesses of each classification model, offering valuable insights for future improvements [26]. A comparative analysis of the models used in this study is presented in Fig. 5.

**Fig. 6:**
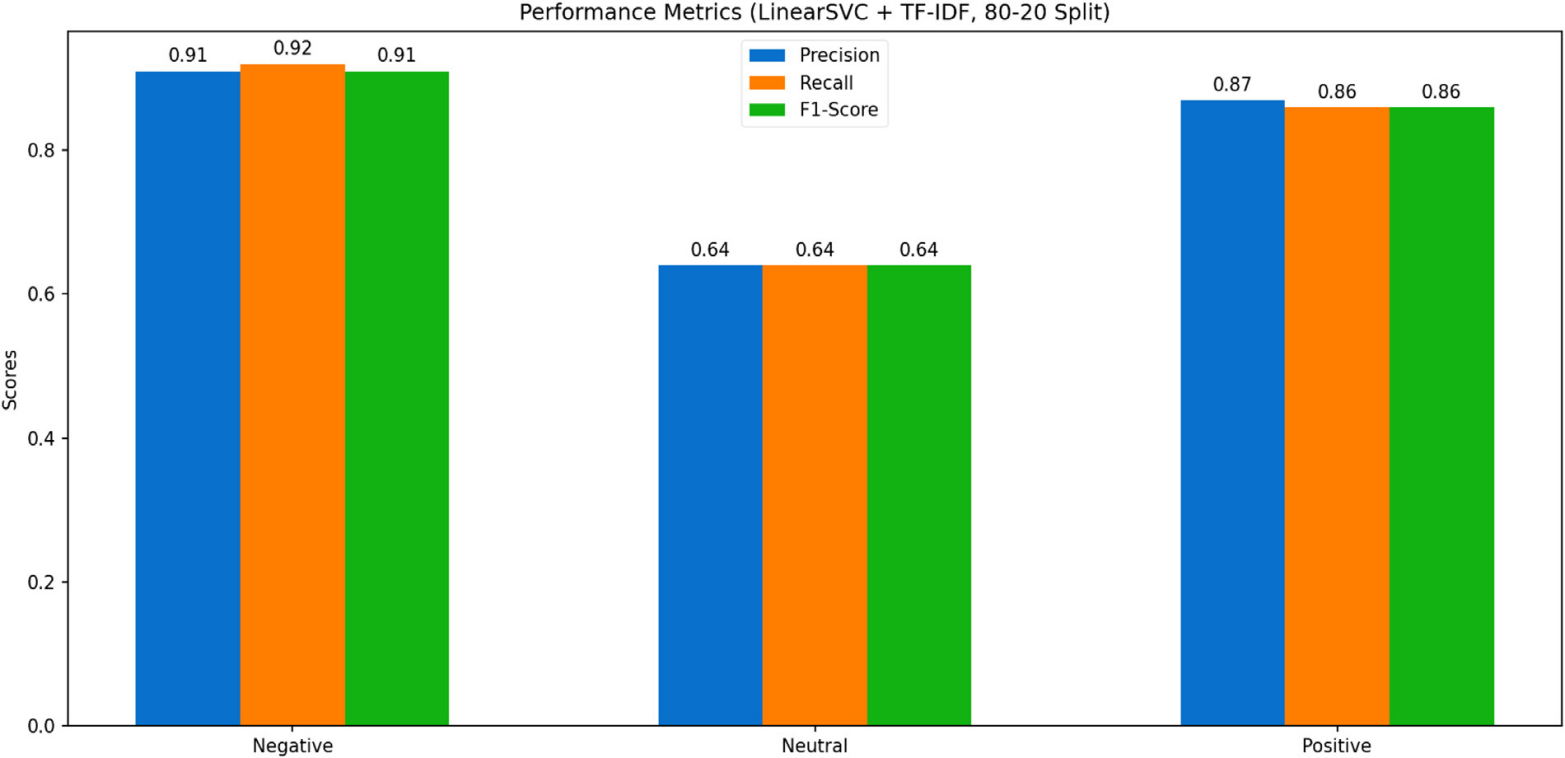
Performance of Linear SVC.

The analysis of performance metrics for the selected model is presented in Fig. 6. The comparative evaluation of machine learning models for sentiment analysis, specifically in the context of vaccination-related tweets, highlights several key findings. The evaluation metrics, particularly the components of the confusion matrix (true positives, false positives, and false negatives), reveal variations in model performance across different sentiment classes—Negative, Neutral, and Positive. These results provide insights into the effectiveness of different feature combinations and models in accurately classifying sentiment [27].

**Confusion Matrix** is a performance measurement tool for classification models, providing a visual representation of how well the model performs across different classes. It summarizes the count of true positives (TP), true negatives (TN), false positives (FP), and false negatives (FN) in a matrix format, allowing researchers and practitioners to quickly assess the classification results. In the context of classification, the confusion matrix is particularly useful because it highlights not only the overall accuracy of the model but also its performance on individual classes. This granularity helps identify which classes the model is performing well on and which ones may require further improvement or attention.

### Key metrics derived from the confusion matrix

**Accuracy** is the ratio of correctly predicted instances (both true positives and true negatives) to the total number of instances. It provides a general measure of the model's overall performance.

$$\text{Accuarcy}=\frac{\text{TP}+\text{TN}}{\text{TP}+\text{TN}+\text{FP}+\text{FN}}$$

**Precision** indicates the accuracy of positive predictions. It measures the proportion of true positive predictions relative to the total number of positive predictions (true positives plus false positives). High precision indicates that a model has a low false positive rate [28].

$$\text{Precision}=\frac{TP}{TP+FP}$$

**Recall** also known as sensitivity or true positive rate, recall measures the proportion of actual positives that were correctly identified by the model. It reflects the model's ability to capture all relevant instances.

$$\text{Recall}=\frac{\text{TP}}{\text{TP}+\text{FN}}$$

**F1-Score** is the harmonic mean of precision and recall, providing a balance between the two metrics. It is particularly useful when the class distribution is imbalanced, as it takes both false positives and false negatives into account.

$$F1-\text{score}=2\times \frac{\text{Precision}\times \text{Recall}}{\text{Precision}+\text{Recall}}$$

These measures, when combined with the confusion matrix, provide a thorough knowledge of the classification model's performance, highlighting both its advantages and disadvantages to guide future developments [29].

**Fig. 7:**
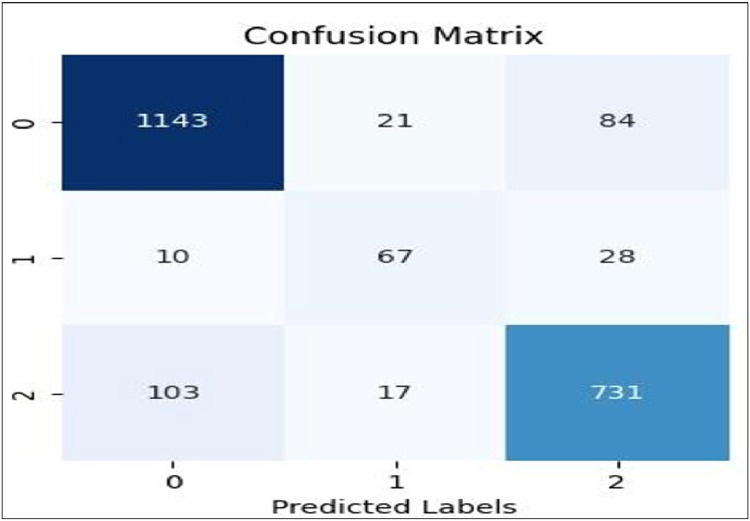
Linear SVC confusion matrix.

**Fig. 8:**
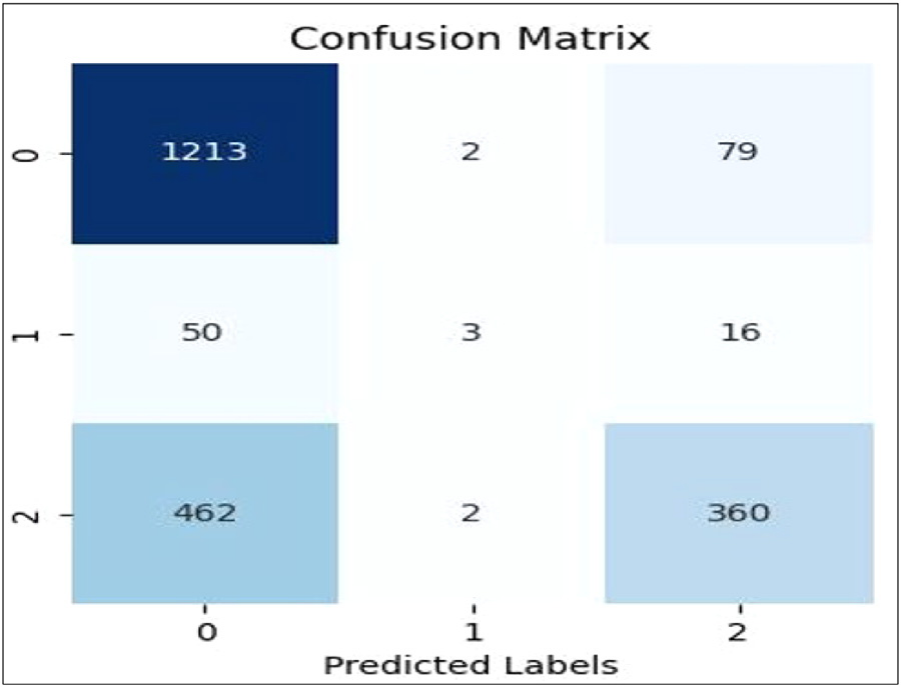
GBM confusion matrix.

**Fig. 9:**
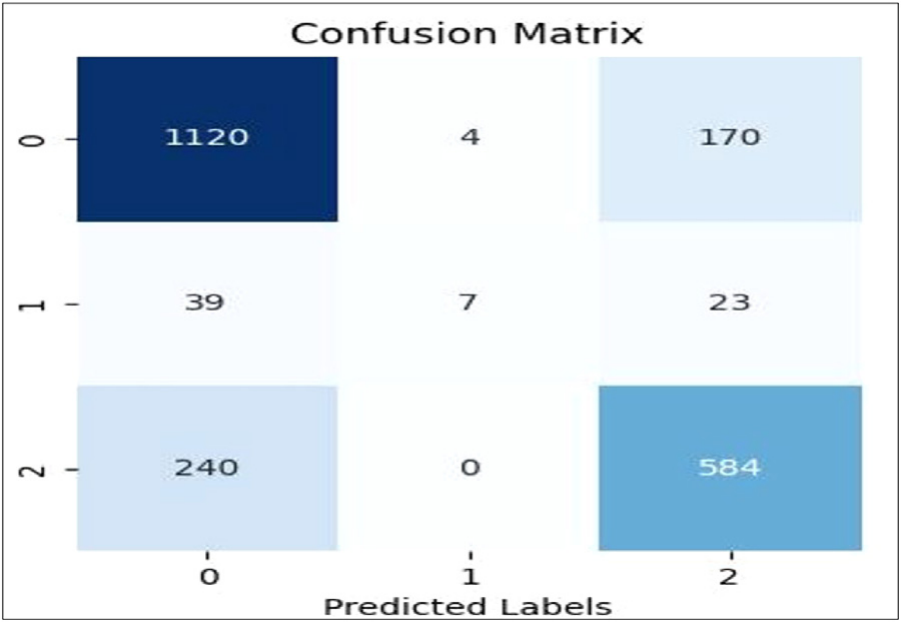
RF confusion matrix.

**Fig. 10:**
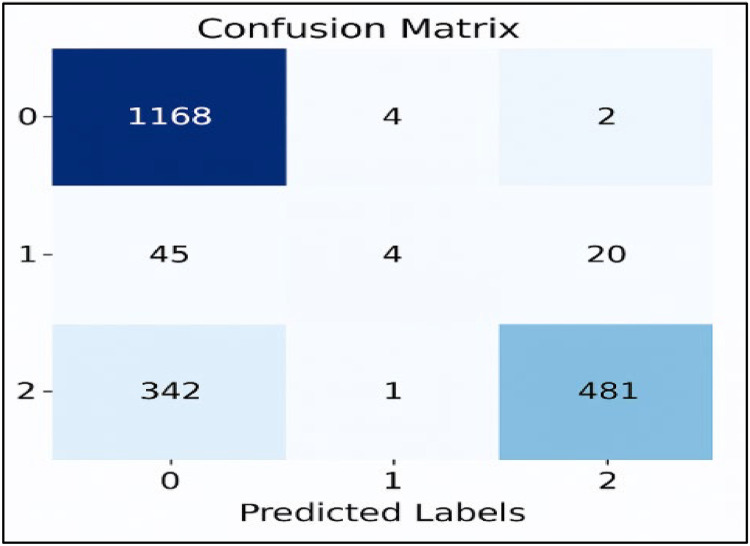
GBM confusion matrix.

**Fig. 11:**
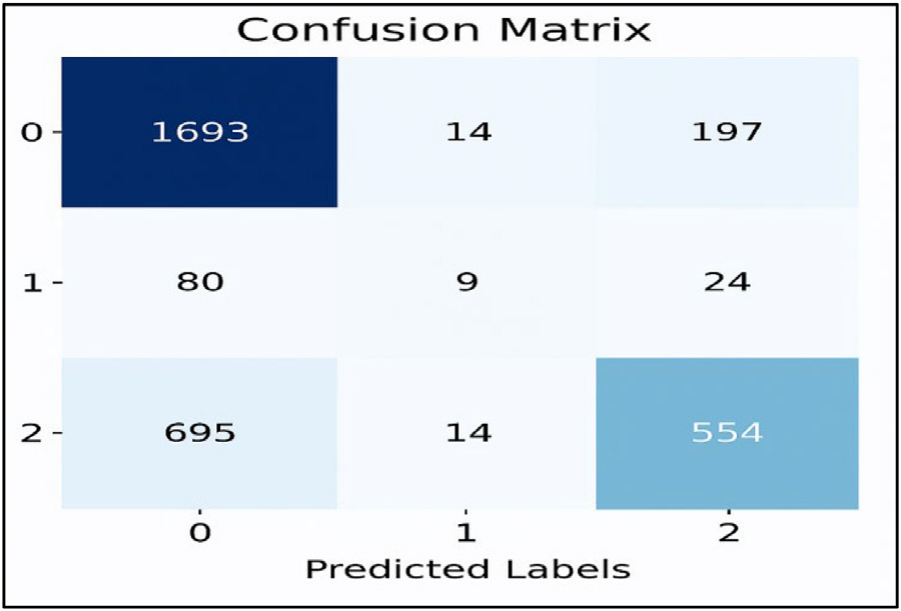
AdaBoost confusion matrix.

Fig. 7, Fig. 8, Fig. 9, Fig. 10, Fig. 11 show the confusion matrices for Linear SVC, GBM, Random Forest, XGBoost, and AdaBoost, respectively. Each confusion matrix was generated using an 80:20 train-test split and TF-IDF feature extraction. Here, class ‘0’ denotes negative sentiment, ‘1’ neutral, and ‘2’ positive. In each matrix, rows represent actual classes and columns predicted ones, with diagonal cells indicating correct predictions. Among all models, Linear SVC achieved the highest accuracy and F1-score, effectively distinguishing among all sentiment categories, while ensemble models showed varying degrees of performance, especially in classifying neutral sentiments.

Table 1 compares the performance of various ML models (Linear SVC, Random Forest, Gradient Boosting Machine, XGBoost and AdaBoost) for sentiment analysis of vaccination-related tweets, using two feature extraction methods: TF-IDF and Word2Vec. The results, evaluated on two different train-test splits (70–30 and 80–20), show that TF-IDF-based models generally outperform Word2Vec models [30] achieving higher accuracy and better performance in classifying Negative and Positive sentiments. For instance, Linear SVC with TF-IDF achieved the highest accuracy of 88.07 %, while models using Word2Vec showed lower accuracy, particularly for Neutral sentiment classification.

**Table 1:** Tabular representation of performance obtained from ML models for sentiment analysis.

Model Embedding	Train -Test Split	Accuracy	Negative Sentiments			Neutral Sentiments			Positive Sentiments		
			Precision	Recall	F1 Score	Precision	Recall	F1 Score	Precision	Recall	F1 Score
**Linear SVC +TF-IDF**	70–30	86.75	0.91	0.90	0.90	0.89	0.86	0.80	0.84	0.85	0.85
**Linear SVC +TF-IDF**	**80–20**	**88.07**	**0.91**	**0.92**	**0.91**	**0.85**	**0.85**	**0.85**	**0.90**	**0.89**	**0.89**
**Linear SVC +W2V**	70–30	66.00	0.68	0.82	0.74	0.12	0.01	0.02	0.61	0.46	0.53
**Linear SVC +W2V**	80–20	65.00	0.68	0.84	0.80	0.50	0.04	0.08	0.68	0.54	0.54
**RF+TF-IDF**	70–30	77.00	0.79	0.84	0.81	0.82	0.12	0.22	0.73	0.71	0.72
**RF+TF-IDF**	80–20	78.00	0.80	0.87	0.83	0.64	0.10	0.17	0.75	0.71	0.73
**RF+TF-W2V**	70–30	65.00	0.66	0.84	0.74	0.93	0.12	0.22	0.62	0.41	0.50
**RF+W2V**	80–20	66.00	0.67	0.85	0.75	1.00	0.10	0.18	0.62	0.41	0.49
**GBM+TF-IDF**	70–30	72.00	0.70	0.93	0.80	0.67	0.07	0.13	0.78	0.45	0.58
**GBM+TF-IDF**	80–20	72.00	0.70	0.94	0.80	0.43	0.4	0.08	0.79	0.44	0.56
**GBM+W2V**	70–30	64.00	0.65	0.85	0.74	0.83	0.09	0.16	0.61	0.37	0.46
**GBM+W2V**	80–20	64.00	0.65	0.85	0.74	0.71	0.07	0.13	0.58	0.35	0.43
**XGBoost + *T* F-IDF**	70–30	75.00	0.74	0.90	0.81	0.69	0.08	0.14	0.78	0.59	0.67
**XGBoost +TF-IDF**	80–20	76.00	0.75	0.90	0.82	0.44	0.06	0.10	0.77	0.58	0.66
**XGBoost+ W2V**	70–30	66.00	0.68	0.80	0.74	0.93	0.12	0.22	0.62	0.50	0.55
**XGBoost+ W2V**	80–20	66.00	0.69	0.80	0.74	1.00	0.10	0.18	0.59	0.48	0.53
**AdaBoost+ TF-IDF**	70–30	68.78	0.69	0.89	0.77	0.24	0.08	0.12	0.71	0.44	0.54
**AdaBoost +TF-IDF**	80–20	69.00	0.69	0.89	0.78	0.17	0.04	0.07	0.70	0.42	0.53
**AdaBoost+ W2V**	70–30	61.00	0.63	0.83	0.71	0.50	0.01	0.02	0.54	0.33	0.41
**AdaBoost+ W2V**	80–20	62.00	0.64	0.84	0.73	0.20	0.06	0.09	0.54	0.31	0.40

## Performance evaluation

The performance of the models and embedding techniques was evaluated across various configurations, with the Linear Support Vector Classifier (SVC) paired with Term Frequency-Inverse Document Frequency (TF-IDF) emerging as the most effective combination. Using an 80–20 train-test split, this configuration achieved the highest accuracy of 88.07 %, along with balanced and consistent results across sentiment categories. The F1 scores for negative, neutral, and positive sentiments were 0.91, 0.85, and 0.85, respectively, indicating strong classification capabilities, especially for noisy social media data.

On the other hand, models utilizing Word2Vec (W2V) embeddings generally underperformed, with accuracy dropping to as low as 64 % in some cases, such as in the Gradient Boosting Machine (GBM) with W2V. Moreover, the F1 scores for neutral and positive sentiments often fell below 0.6, suggesting that W2V struggled to capture the subtleties of sentiment, especially for neutral expressions.

When comparing Random Forest (RF) and GBM models, both exhibited similar accuracy rates (around 72 %) when paired with TF-IDF embeddings. However, differences emerged in their performance at the class level. Random Forest showed slightly higher recall for positive sentiments, while GBM exhibited marginally better precision. These variations may influence the model choice depending on the specific application. For example, in situations where false negatives could have significant consequences, such as public health sentiment analysis, prioritizing recall might be more important.

Regarding the influence of the train-test split, the 80–20 split generally resulted in slightly higher accuracy than the 70–30 split, thanks to the larger training data. However, the differences in class-wise F1 performance between the two splits were minimal, suggesting that the models were fairly consistent across both configurations.

Throughout all the models and embedding techniques, negative sentiments were the most accurately classified, frequently achieving F1 scores above 0.7. Positive sentiments also performed well, but detecting neutral sentiments proved more challenging. W2V- model struggled with neutral sentiment, where F1 scores dropped to as low as 0.02. This trend highlights the complexity of identifying neutrality in social media content and underscores the need for more advanced embedding strategies.

**Fig. 12:**
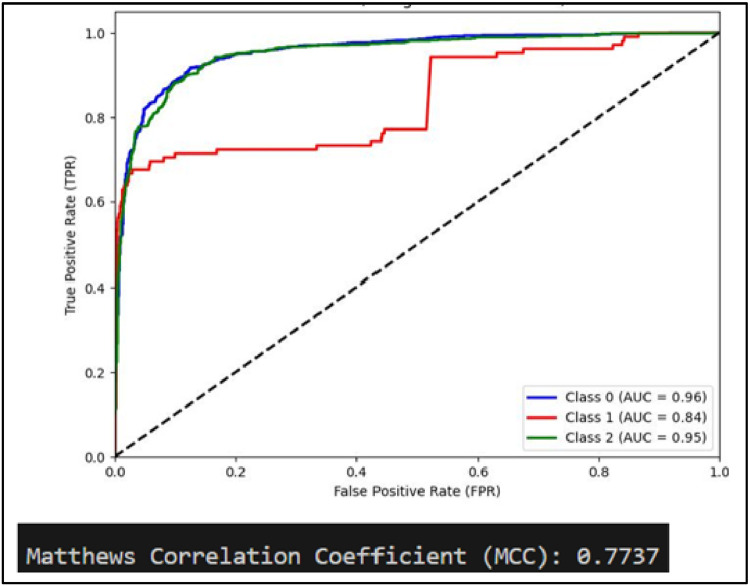
AUCROC curve for linear SVC +TF-IDF.

**Fig. 13:**
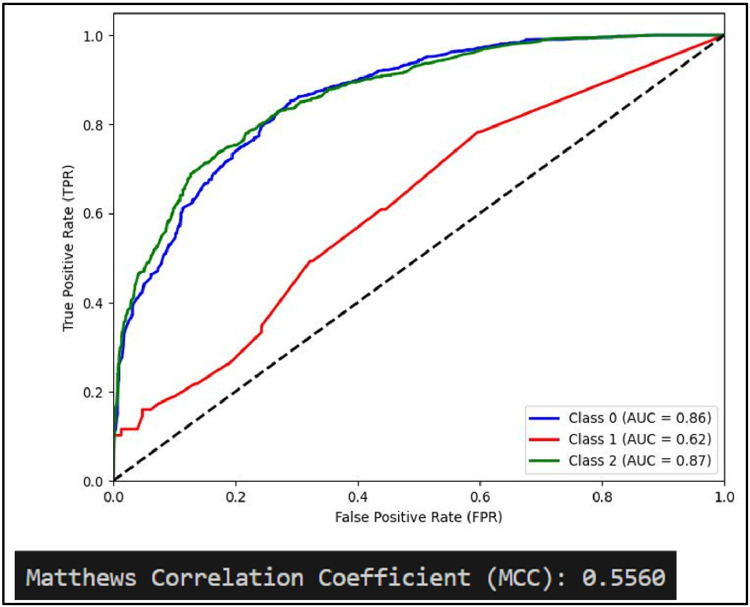
AUCROC curve for RF+TF-IDF.

Another performance measure of a classification model is ROC curve which is a plot of True Positive rate(TPR) against False Positive Rate (FPR) [31]. It is to be noted that if ROC curve hugs to the top left corner of the plot, this shows that the model whose plot is done has higher TPR than FPR. Such model performs well compared to others which are not so. Along with this AUC showing area under the curve quantifies performance of a classification model, the greater the area under the curve, the better model will perform. Plots of AUCROC curve for the models utilized for classification of sentiments is obtained with Train-Test split of 80:20 and TF-IDF being used for feature extraction as both these give better performance. Fig. 12 showing AUC ROC curve for Linear SVC clearly shows more AUC as compared to other models . This signifies Linear SVC is better able to distinguish between the classes and classify them correctly. Further Fig. 13 illustrates that RF is next in line after SVC for classification of Positive and Negative classes but it performs poor in classification of Neutral sentiments.

**Fig. 14:**
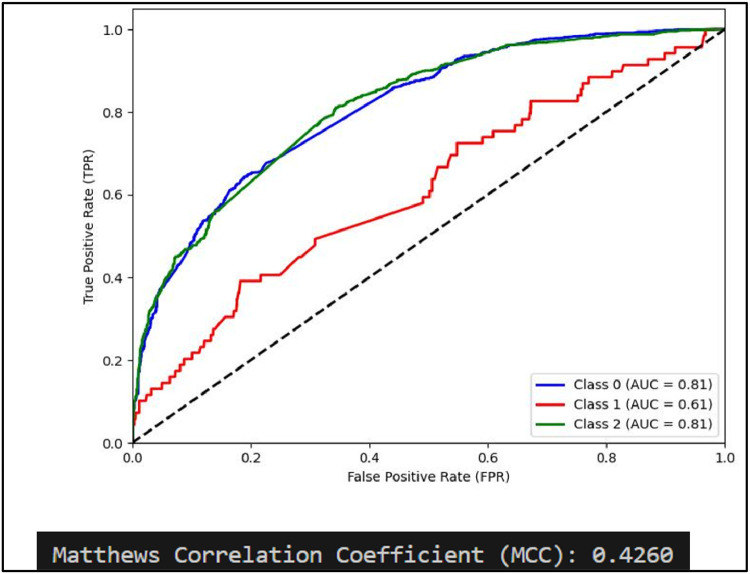
AUCROC curve for GBM + TF-IDF.

**Fig. 15:**
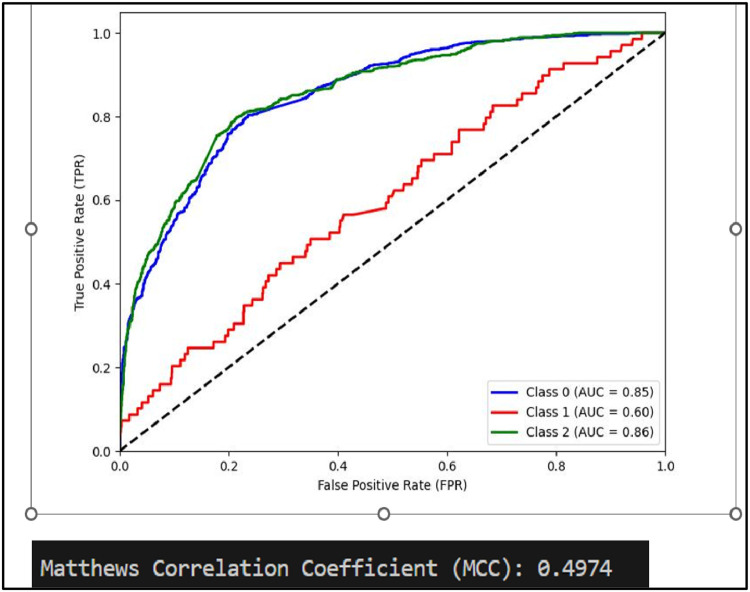
AUCROC curve for XGBoost + TF-IDF.

**Fig. 16:**
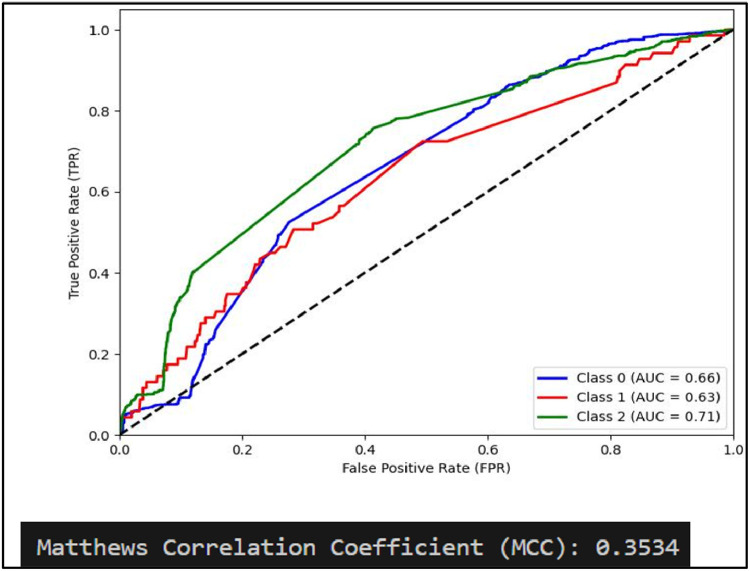
AUCROC curve for AdaBoost + TF-IDF.

Mathew Correlation Coefficient(MCC) is another statistical tool used for performance evaluation of a model. It is a measure of difference between actual and predicted values and is considered as a balance measure that can be used to assess performance even if classes are of different sizes. Along with AUCROC curve model MCC is also considered with Linear SVC giving the highest measure as is clear from Fig. 12. AUCROC plot of GBM (Fig. 14) and XGBoost (Fig. 15), though seems to be satisfactory, but MCC values of both models are below 0.5 reflecting a weak correlation between actual and predicted sentiments. Fig. 16 showing AUCROC plot and MCC value for AdaBoost model highlighting that the model suffers to differentiate between classes . Also, MCC value is least among all the models selected signifying Adaboost lacks to distinguish between sentiments associated with the text used.

**Fig. 17:**
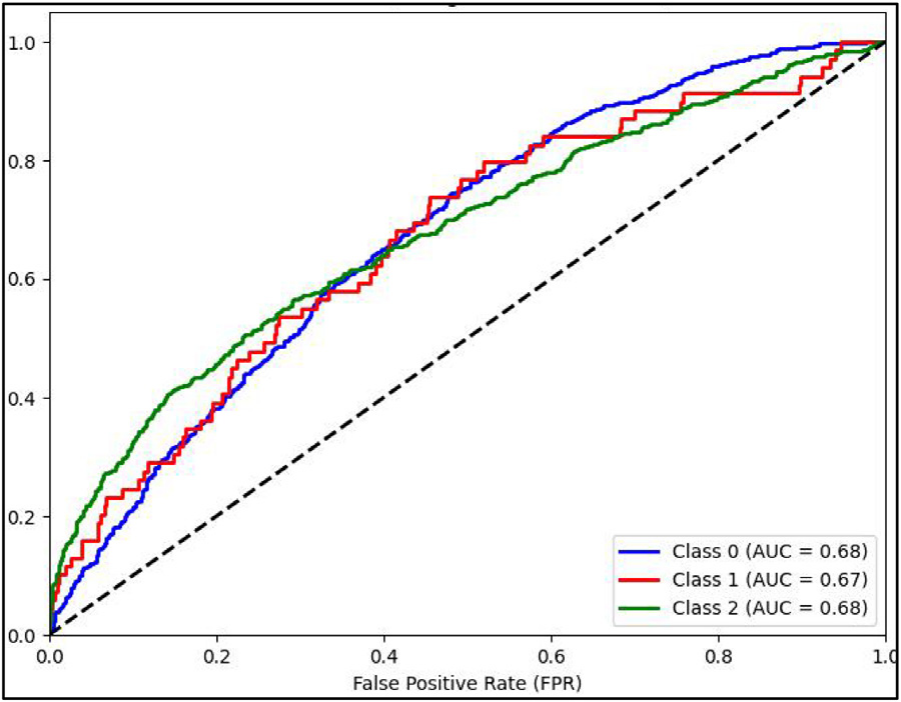
AUCROC curve for linear SVC +W2V.

**Fig. 18:**
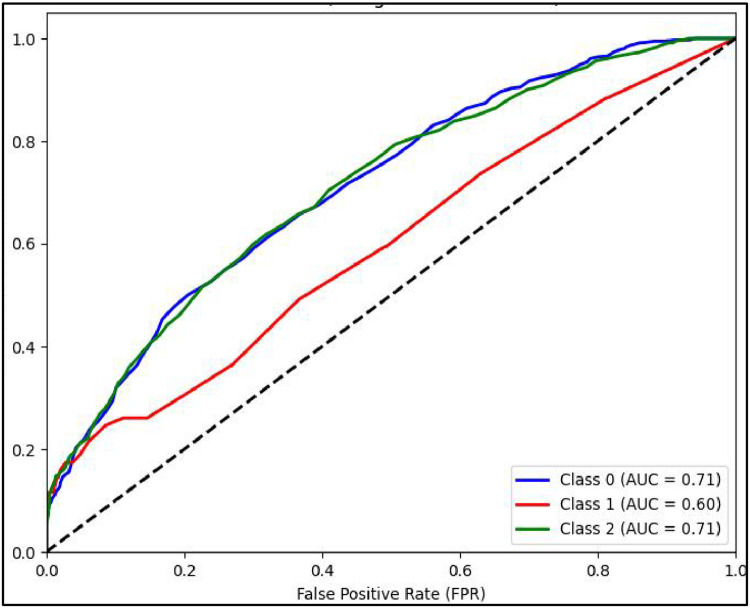
AUCROC curve for RF +W2V.

**Fig. 19:**
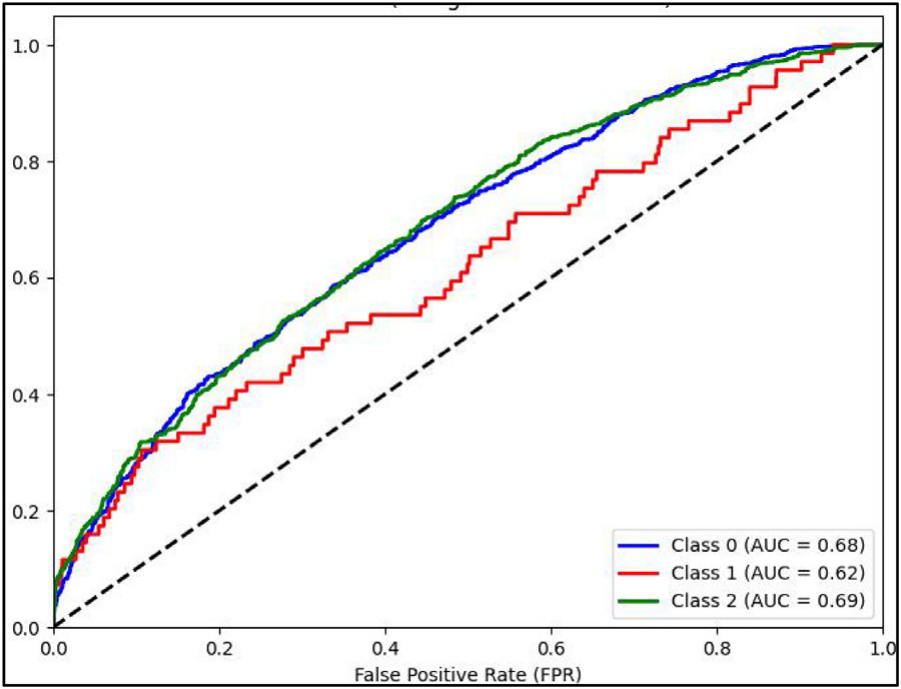
AUCROC curve for GBM +W2V.

**Fig. 20:**
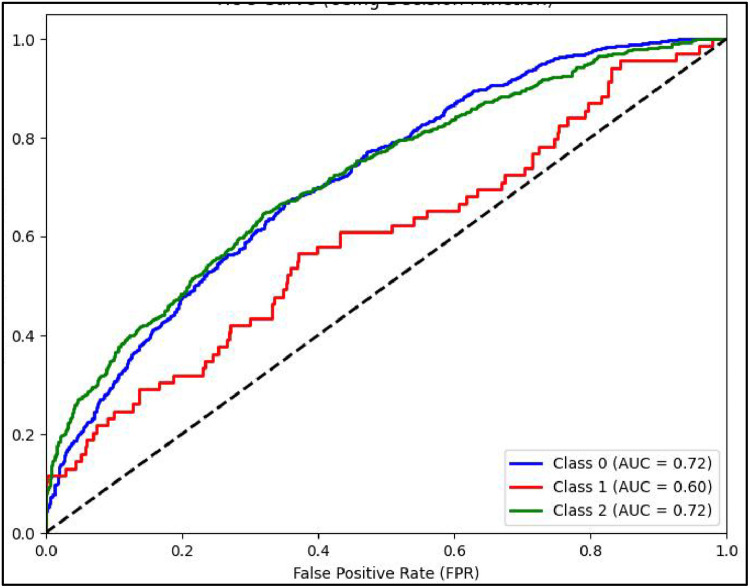
AUCROC curve for XGBoost +W2V.

**Fig. 21:**
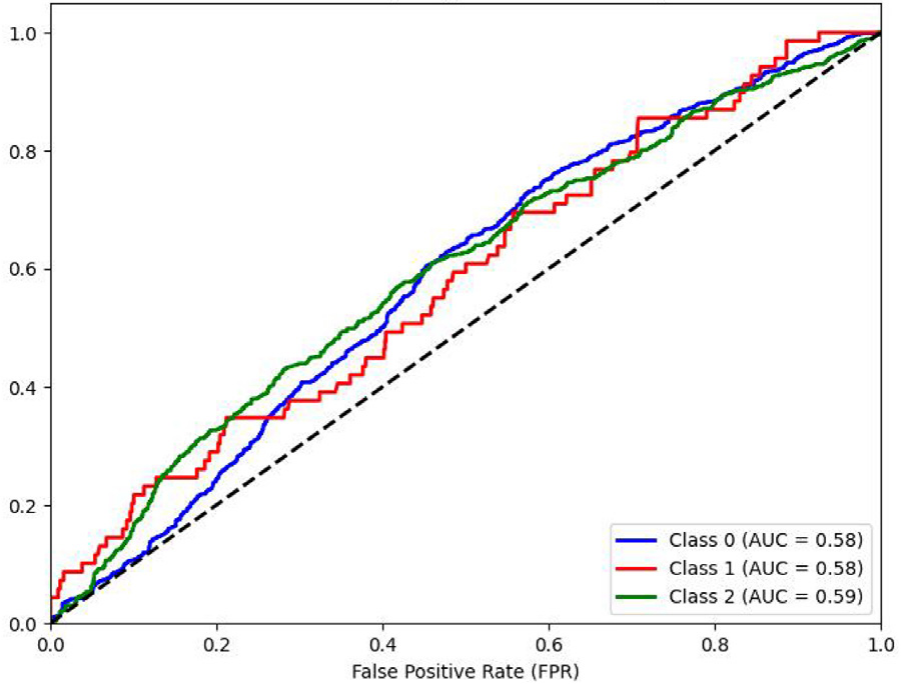
AUCROC curve for AdaBoost +W2V.

Plots of AUCROC curve for the models utilized for classification of sentiments is obtained with Train-Test split of 80:20 and utilizing W2V for feature extraction are shown from Fig. 17, Fig. 18, Fig. 19, Fig. 20, Fig. 21. As is clear from the Fig. 17 for the plot of curve for Linear SVC the model curve remains same for all three class of sentiments. Fig. 18 shows AUCROC curve for RF ensemble model with W2V used for feature extraction. On comparison of same model with TF-IDF embedding model, with W2V the performance od model for sentiment classification is poorer as with TF-IDF embedding the model works very well with class 0 and Class2 viz. with negative and positive sentiments.

Fig. 19 shows the performance matrices AUCROC curve for GBM model, it can be inferenced that for all the three class of sentiments the model performs equally but still not as good as when combined with another embedding TF-IDF technique. Fig. 20 shows the curve for XGBoost model. It can be inferred that for positive and negative class for both types of word embedding(TF-IDF and W2V) with all ML model respond better than with neutral class.

Fig. 21 shows plot of AUCROC curve for Adaboost model with W2V used for model embeddings with 80:20 split of Training and Test data. The model though performs poorly compared to TF-IDF embedding but able to differentiate all three classes fairly the same.

## Conclusion

This study demonstrates the effectiveness of integrating advanced natural language processing (NLP) models, rigorous preprocessing, and feature engineering to address the complexities of sentiment analysis, particularly for vaccination-related tweets. The DistilBERT model, fine-tuned on SST-2, played a pivotal role in achieving accurate sentiment classification, offering a balance between computational efficiency and nuanced language understanding. The labeled dataset generated through the pipeline enabled rigorous evaluations of machine learning models, including LinearSVC, Random Forest, and GBM, each of which exhibited unique strengths in addressing sentiment classification tasks. The sentiment analysis was performed using various machine learning models, combined with TF-IDF and Word2Vec embeddings. The results revealed that LinearSVC with TF-IDF embeddings achieved the highest accuracy, with the 80–20 train-test split showing an accuracy of 88.07 %, outperforming other combinations. Additionally, the models could benefit from an ensemble approach, where multiple models are combined, to reduce misclassifications and enhance the overall performance. The ensemble technique has been shown to improve accuracy and robustness by leveraging the strengths of different models.

In addressing the core objectives of this study, several key contributions were made. First, the application of DistilBERT for pseudo-labeling significantly improved sentiment classification in noisy, unlabeled Twitter data, capturing semantic nuances that traditional models often overlook. Second, the comparative evaluation of TF-IDF and Word2Vec embeddings across various classifiers revealed that TF-IDF combined with LinearSVC consistently outperformed other configurations, establishing it as the most effective pipeline. Lastly, the study highlighted the limitations of existing methods in accurately identifying neutral sentiments and demonstrated that a hybrid approach—integrating transformer-based models with conventional techniques—offers a promising direction for improving classification in this challenging category.

Despite the promising results, it is important to acknowledge certain limitations of this study. One significant boundary condition is the influence of ideological factors and widespread misinformation, particularly in regions where vaccine hesitancy has resurged following the COVID-19 pandemic. Moreover, the politically polarized nature of online discourse can shape the tone and content of tweets, introducing biases in the data itself. As a result, sentiment patterns derived from such datasets may reflect dominant narratives or ideological leanings rather than neutral public opinion. While sentiment analysis provides valuable insights into public attitudes, it cannot fully capture or counter deep-rooted ideological resistance to vaccination. In countries where skepticism toward vaccines has led to the reemergence of previously eradicated diseases, targeted public health campaigns must complement technological solutions, emphasizing scientifically grounded messaging to confront misinformation effectively. Future work should consider integrating ideological analysis and misinformation tracking to enhance the robustness and practical impact of sentiment-based insights in public health initiatives.

In future work, incorporating these advanced methods could significantly increase the reliability of the sentiment analysis pipeline, providing more accurate results for nuanced sentiment categories like Neutral. These advancements are especially crucial in fields like public health communication, where understanding public sentiment is essential for effective decision-making and outreach. By improving sentiment classification, policymakers and health organizations can better engage with the public and tailor communication strategies to address concerns and promote positive vaccination attitudes.

## Limitations

Despite the promising results, several limitations were identified, notably the difficulty in accurately classifying Neutral sentiments. This limitation suggests that incorporating advanced techniques, such as contextual embeddings like BERT or DistilBERT, could help improve the model's ability to capture subtle sentiment variations. Also for present sentiment analysis of Covid Vaccine tweets, data used is from a single source. Inclusion of diverse datasets from different sources will ascertain model generalizability of sentiment classification at times of pandemic and others.

## Ethics statements

Participant data has been fully anonymized to ensure privacy, and the data redistribution policies of Kaggle have been fully complied with, adhering to ethical guidelines for data usage.

## CRediT authorship contribution statement

**Renuka Agrawal:** Conceptualization, Validation, Supervision. **Mehuli Majumder:** Investigation, Resources, Writing – review & editing. **Ishita Yadav:** Investigation, Resources, Writing – original draft. **Nandini Taneja:** Data curation, Formal analysis, Visualization. **Safa Hamdare:** Writing – review & editing, Validation. **Preeti Hemnani:** Writing – review & editing.

## Declaration of competing interest

The authors declare that they have no known competing financial interests or personal relationships that could have appeared to influence the work reported in this paper.

## Data Availability

Data will be made available on request.
